# Supplementary material for: Qualitative Exploration of the ‘Rolling Unmasking Effect’ for Downwind Odor Dispersion from a Model Animal Source
Source: Int J Environ Res Public Health. 2021 Dec 11;18(24):13085. doi: 10.3390/ijerph182413085 (PMC8702010; doi:10.3390/ijerph182413085)
Supplement: Supplementary file 1 [file ijerph-18-13085-s001.zip › ijerph-1426694-supplementary.pdf]

Supplementary Material

Qualitative Exploration of the 'Rolling Unmasking Effect' for Downwind Odor Dispersion from a Model Animal Source

Donald W. Wright [1,*], Jacek A. Koziel [2,*], David B. Parker [3], Anna Iwasinska [4], Thomas G. Hartman [5], Paula Kolvig [6] and Landon Wahe [7]

[1] Don Wright & Associates, LLC, Georgetown, TX 78628, USA
[2] Department of Agricultural and Biosystems Engineering, Iowa State University, Ames, IA 50011, USA
[3] College of Engineering, West Texas A&M University, Canyon, TX 79016, USA
[4] Volatile Analysis Corporation Inc., Grant, AL 78664, USA
[5] Department of Food Science, Rutgers University, New Brunswick, NJ 08901, USA
[6] Moody Gardens, Galveston, TX 77554, USA
[7] Department of Civil, Construction, and Environmental Engineering, Iowa State University, Ames, IA 50011, USA
* Correspondence: dwrigh256@gmail.com (D.W.W.); koziel@iastate.edu (J.A.K.);
Tel.: +1-512-750-1047 (D.W.W.); +1-515-294-4206 (J.A.K.)

Details of Methods

P.T. Porcupine; urine collection (Paula Kolvig; Moody Gardens Rainforest Exhibit)

At the request of the panelist (D.W.W.), the Moody Gardens Rainforest Exhibit staff carried out a passive, external urine collection from Bono, the 10-year-old male half of the Moody Gardens breeder pair. The urine sample collection was carried out on April 12, 2017, utilizing a passive / 'dirty' collect procedure, which allowed for some mixing of fecal solids with the liquid urine sample. As explained to the Rainforest Exhibit staff, this procedure enabled the panelist (D.W.W.) to execute a qualitative odor profile assessment of the combined waste from the male P.T. porcupine, although the urine was projected as the major reservoir for the targeted 'onion' odor.

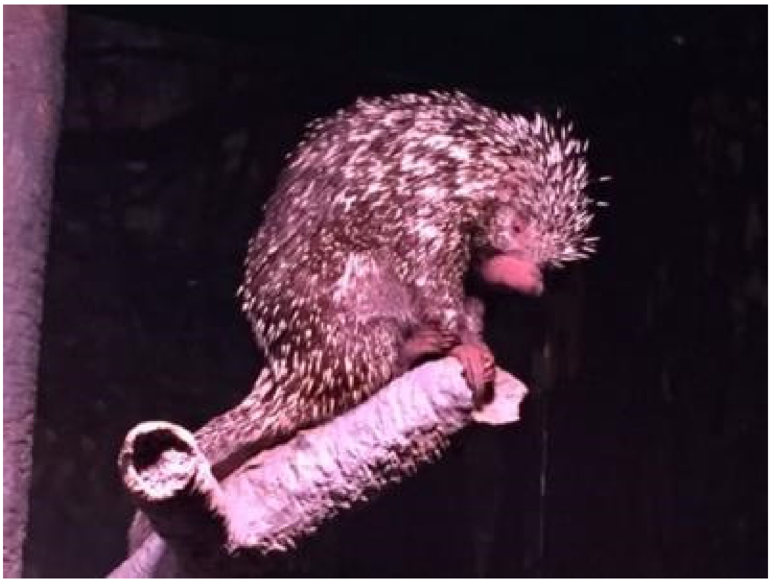

Figure S1. Cora; female porcupine at Moody Gardens, Galveston, Texas (photo by D.W.W., [1]).

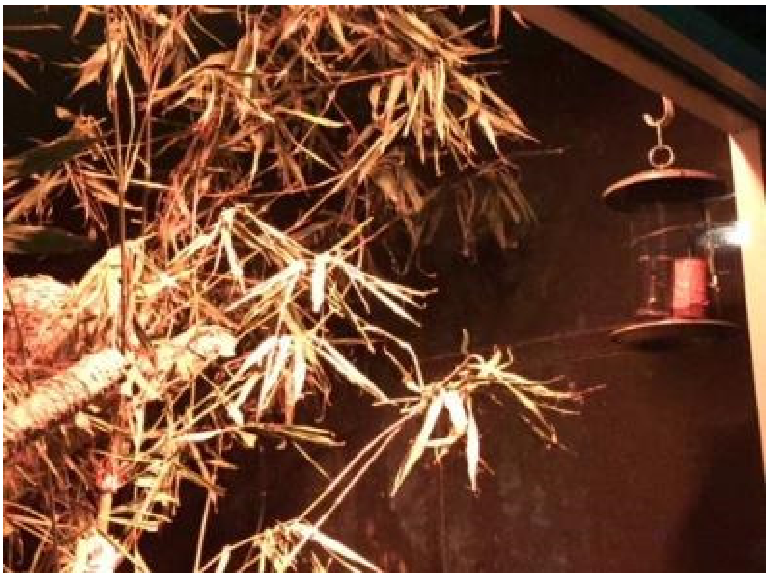

Figure S2. Sampling point at a porcupine exhibit chamber at Moody Garden. SPME fibers protected by affixing to hanging fixture (photo by D.W.W., [1]).

P.T. Porcupine; Chemical analyses by collaborative investigators

Collaborative Investigator #1: (Thomas G. Hartman, Rutgers University)

Beginning with about 2 mL of PT porcupine urine provided, it was saturated with NaCl; extracted into 5 mL of diethyl ether; centrifuged; lifted off the ether extract and then evaporated 1/2 of the ether extract into the purge & trap apparatus (in duplicate). The sample extract was then purged with nitrogen for 30 min at 100 °C onto Tenax TA traps. The Tenax TA traps were thermally desorbed into the GC-MS at 250 °C·5 min^{-1}. The GC was cryogenically temperature programmed from minus 20 °C (5 min) during desorption, followed by 10 °C·min^{-1} to 280 °C. The column was a single 60 m x 0.32 mm id x 1.0 um film ZB5MS. The mass spectrometer, a Thermo-Finnigan Mat TSQ-7000, was set to scan the mass range 35-350 amu once per second. The data files were converted to HP Chemstation format using MassTransit software and burned onto a CD so they could be processed on the panelist (D.W.W.) Chemstation / Benchtop PBM system. The original data format was Thermo Xcaliber. A background plot was plotted (i.e., mass chromatogram 35_350-40-44) to background-subtract argon and CO_2 to clean up the chromatograms when viewing.

The urine and ether extracts were sniffed before and after pre-concentration and confirmed to retain a strong odor in the extract delivered to the purge and trap apparatus. The Solid Sample Purge & Trap Collection System and Model TD-4 Short Path Thermal Desorber (i.e., both from Scientific Instrument Services; SIS, Ringoes, NJ) was the integrated system utilized for volatiles pre-concentration. Short Path Thermal Desorption (i.e., SPTD, a technology for which Dr. Hartman holds the utility patent) utilized Tenax TA packed traps to pre-concentrate the extracted VOCs. The samples were run in duplicate, extending one of the runs to get a few high boilers, so there were 3 pre-concentrated runs total. The spectral data was picked through, scan by scan, but unfortunately, no sulfur compounds stood out for either Dr. Hartman or for the panelist (D.W.W.) in the subsequent data cross-check effort. Although these efforts failed to identify the two targeted 'grilled onion' odorants, they did serve as cross-check identity confirmation for key semi-volatile odorants of secondary 'barnyard' character-impact (e.g., *p*-cresol, 4-ethyl phenol, indole, skatole).

Collaborative Investigator #2: (Anna Iwasinska; Volatile Analysis Corporation)

An Agilent 5975B based AromaTrax™ system (i.e., generally applied as described above for panelist (D.W.W.) was operated in MS-SCAN mode for targeted odorant identification/confirmation. Significant deviations from the parameters listed for the panelist (D.W.W.) were: Column # 1: 30 m x 0.53 mm ID BPX 5 – 0.5μm film (pre-column, from SGE); Column # 2: 30 m x 0.53 mm ID SolgelWAX – 0.1 μm film (analytical column, from SGE). The chromatographic data were acquired using Agilent MSD ChemStation Data Acquisition software (E.02.01.1177). The MSD scan mass range was: 35 amu to 400 amu at a rate of 3.84 scan · sec^{-1}. The analysis of resulting chromatograms was performed using ChemStation Data Analysis software (F.01.03.2357) referencing the Wiley9-N08, NIST11, and FFNSC13 Mass Spectral libraries. The compound identities were reported based on best-match ranking of the experimental spectra against these databases.

The samples of PT porcupine urine and Virginia pepperweed (same batch of materials that was used by panelist) were analyzed by this investigator in order to further explore identification of some leading odor notes. The headspace volatiles collection was conducted in the manner described above for the panelist. The spectral data was reviewed in detail but, despite best efforts, failed to identify the two targeted 'onion' odorants from PT porcupine urine headspace. However, separate spectral interpretive efforts were also applied, by this investigator, to the prairie verbena data developed by the panelist; serving as an independent cross-check of the proposed VOC / odorant identity profile. This effort included, most notably, (1) the tentative identification of hyacinthin in for the 'floral' note @18.0 min RT; (2) a 'possible' identification of an oxime for the isomer family carrying the 'ether' note @6.0 min RT and (3) independent cross-check confirmation of benzyl mercaptan as the character-defining 'burnt-match' odorant in Virginia pepperweed.

Prairie verbena; Headspace sample collection for odorant prioritization

The preconditioned 1 L glass headspace vessels were filled with freshly harvested blossoms, collected at peak maturity from a dense natural cluster (Georgetown, TX). At the season and time of harvesting, the smell at the odor frontal boundary was characterized as a distinct 'barnyard', identical to that recalled for the pure odorant, *p*-cresol.

Virginia pepperweed; Headspace sample collection for odorant prioritization

The preconditioned 1 L glass headspace vessels were filled with freshly harvested whole plants at peak maturity (i.e., stems + blossoms), reflecting either 'pristine' or 'crushed' conditions. Crushing was necessary to release the characteristic 'burnt match' odor. The plants were harvested from sparse/random distribution within lawn/field environments (Georgetown, TX). When harvesting, the macerated whole plants' smell at the frontal boundary was characterized as a distinct 'burnt match,' identical to that recalled for the pure odorant benzyl mercaptan.

Prairie verbena and Virginia pepperweed; MDGC parameters for odorant prioritization

In contrast to the MDGC based heart-cut isolation protocol, as applied to the 'onion' odorants for the P.T. porcupine urine, both the prairie verbena and Virginia pepperweed headspace VOC / odor profiles were processed in overview survey mode; with total heart-cuts taken between 0.25 min to 32.0 min.

Prairie verbena and Virginia pepperweed; odorant chemical identification

The proposed character-defining odorant identities for both the prairie verbena and Virginia pepperweed were confirmed via GC retention time, odor character matching and mass spectral profile matching.

Details of Results

While the natural-model odor-source selected for focus in this manuscript is the prehensile-tailed porcupine, the authors have also carried out detailed odorant prioritization assessments of two additional natural odor sources from the plant world; prairie verbena and Virginia pepperweed (both indigenous to Central Texas). While not carried through to the same level of validation as the P.T. porcupine they do present additional insight into the odorant prioritization interpretative process. These results are summarized as follows:

Case Study #2: Prairie verbena

Prairie verbena varieties have a wide range of odor characteristics, spanning from virtually odorless (i.e., especially among many commercial hybrids), pleasantly fragrant, to remarkably unpleasant. The focus of this case study is the prairie verbena (i.e., *Verbenaceae*, *Glandularia bipinnatifida*); specifically, the regionally isolated variety of verbena which is native to the boundary region between adjacent Blackland Prairie and Edwards Plateau ecological regions near Georgetown, Texas.

Initial odor assessment at the source and downwind

Upon entering the verbena's odor frontal boundary, the panelist (D.W.W.) was immediately struck by its surprising intensity of familiar 'barnyard' odor (perceived as similar to past studies targeting mammals as environmental odor sources [2-4]). After eliminating obvious CAFO as a source, a closer inspection of the prairie verbena cluster upwind was confirmed as the 'barnyard' odor source (Figures S3-S5).

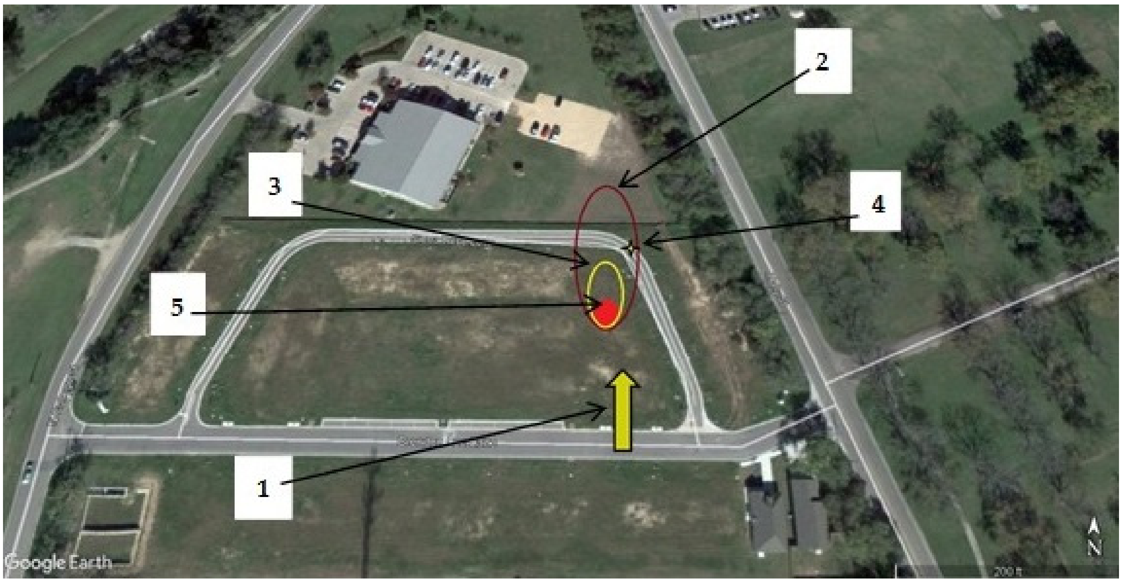

Figure S3. Prairie verbena source of 'barnyard' downwind odor. (1) Wind direction; (2) 'barnyard' odor frontal boundary (red oval); (3) 'floral' secondary (near-source) boundary (yellow oval); (4) investigator's location upon initial encounter in Georgetown, TX and (5) location of odor source cluster of native prairie verbena. Google Earth image.

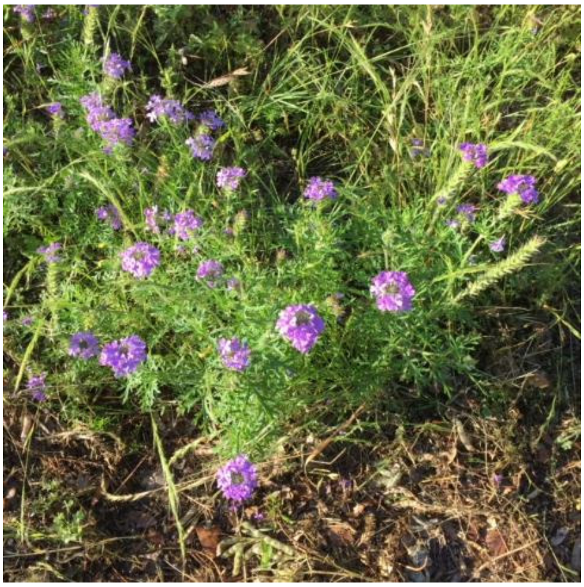

Figure S4. Prairie verbena cluster; *p*-cresol 'barnyard' odor source (photo by D.W.W., [1]).

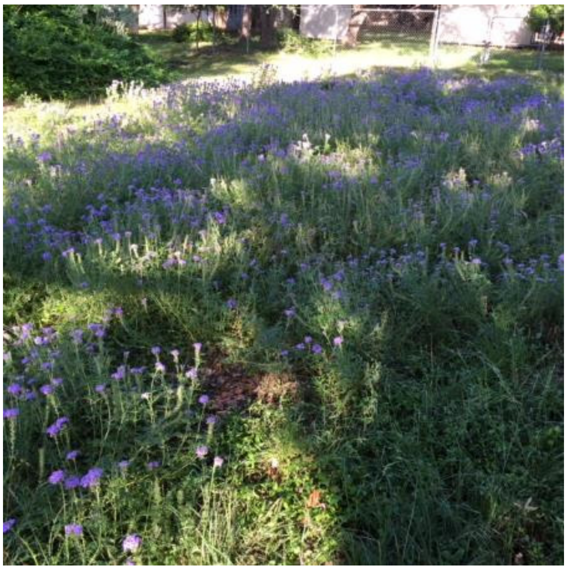

Figure S5. Prairie verbena field; *p*-cresol 'barnyard' odor source (photo by D.W.W., [1]).

Working hypothesis based upon environmental odor assessment

The working hypothesis was: 'due to the remarkable similarity in downwind odor characteristics, there is assumed to be some chemical compound commonality between the priority odorant subsets for the prairie verbena and typical 'agrarian,' sources of 'barnyard' odors.' The driving questions were:

- Are there common character-impact odorants for both prairie verbena and swine CAFO sources that account for the striking similarity in composite odor at their respective odor frontal boundaries?

- What is the overall agreement between the prairie verbena and swine CAFOs when comparing their minimum downwind priority odorant subsets with their full (at-source) odorant and underlying VOC profiles?

MDGC-MS-O based environmental odorant prioritization

The analytical phase focused on the complex VOC emissions (Figure S6, total ion chromatogram) and corresponding odorant profile (Figure S7, aromagram). The volatiles profile (Figure S6) approached 100 discrete compounds even though the initial sampling and sample preparation favored relatively low extraction. As a result, the smallest chromatographic peaks represent concentrations in the high ppt to low ppb range. The complexity is also reflected in the range of chemical functionalities, including terpenes, hydrocarbons (saturated and unsaturated), ketones (aliphatic and aromatic), alcohols (aliphatic and aromatic), esters (aliphatic and aromatic), and phenolics. Likewise, the odorant profile (Figure S7) approaches 50 discrete odor 'notes'.

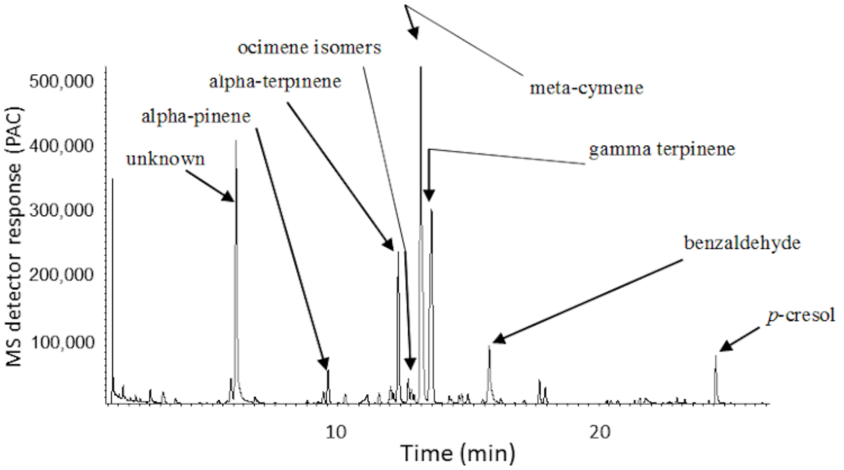

Figure S6. Overview of the prairie verbena headspace volatiles; TIC overview VOC profile, generated in ms-SCAN acquisition mode.

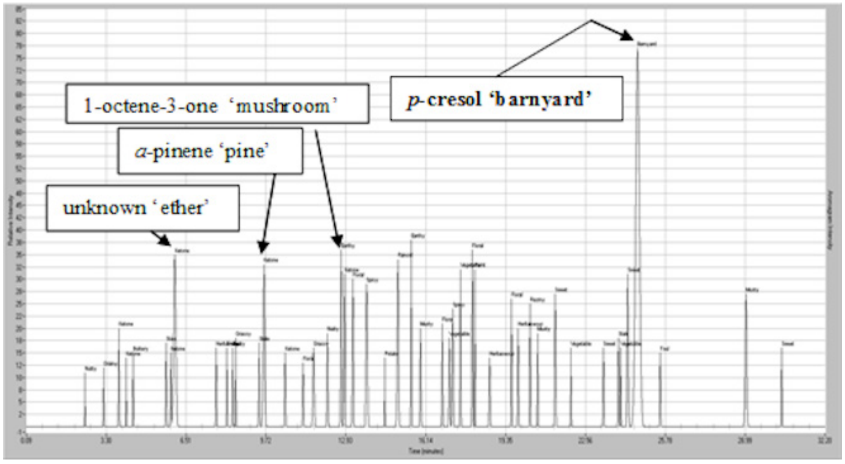

Figure S7. Overview odor profile of the prairie verbena headspace volatiles; aromagram odor profile, generated by a trained panelist evaluating separated odor 'notes' at the sniff port of MD-GC-MS-O.

These ‘modest’ VOC (~100 peaks in Figure S6) and odor note (~50 notes in Figure S7) numbers are certain to increase several-fold if pre-concentration is required to reduce the detection limits from the low ppb to low ppt and below. Such trace-level concentrations are odor-significant for many odorous VOCs. Without direct sensory correlation (Figure S7), the analyst taking the instrument-only approach (Figure S6) is faced with the daunting task of identifying all possible odorants representing potential high-impact and inferring, which ultimately could constitute a character-impact subset. Instrument-only efforts often end in disappointment since the highest-impact odorants are typically at trace concentration levels, often ‘buried’ within a complex background matrix of chromatographic peaks.

Likewise, the lack of critical compositional data (Figure S6) can derail the sensory-only approach at the opposite extreme. Interestingly, among the arguments used for necessitating a sensory-only approach to environmental odor is the analytical complexity. It is often argued that the human olfactory response is inherently complex and, as a result, the composite odor from such complex mixtures is inevitably the ‘combined effect’ of many, if not most, of the odorous VOCs making up the complex emission field. Thus, it might be argued that the characteristic ‘barnyard’ odor, perceived downwind of the prairie verbena source, is the combined response to the ~50 odorants (Figure S7). However, the MDGC-GC-O-based odorant prioritization indicates that this was not the case.

The characteristic ‘barnyard’ odor downwind was perceived as relatively simple; primarily traceable to a single odorant (*p*-cresol). Simplification was achieved using a series of odorant prioritization runs, using SPME exposure time variation to emulate a serial dilution process (i.e., from 70 min ‘undiluted’ reference, 15 min, 5 min, 102 s, 33 s, 11 s, to 3 s). Contrasting VOC / aroma profiles, comparing the ‘dilution’ steps, are shown in Figure S8 (15 min vs. 102 s), Figure S9 (102 vs. 11 s), and Figure S10 (15 min vs. 11 s).

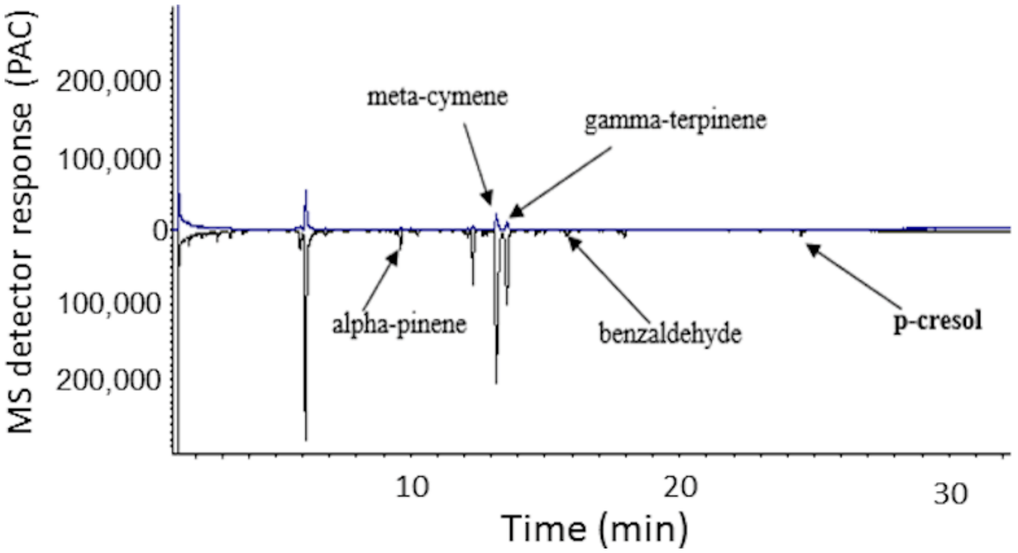

Figure S8. Serial dilution comparisons of the prairie verbena headspace volatiles; TIC VOC profiles, generated in ms-SCAN acquisition mode. Contrasting volatiles collections of 15 min (bottom) and 102 s (top) SPME fiber exposure times. TIC chromatograms are displayed in mirror-image format. *p*-Cresol emerges as <u>one</u> of the key odorants.

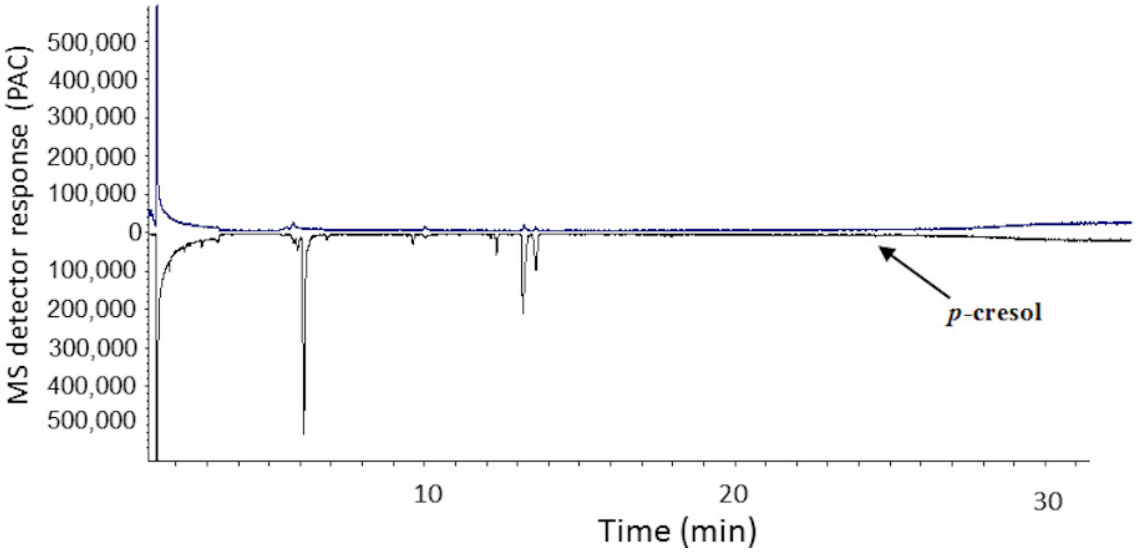

Figure S9. Serial dilution comparisons of the prairie verbena headspace volatiles; TIC VOC profiles, generated in ms-SCAN acquisition mode. Contrasting volatiles collections of 102 s (bottom) and 11 s (top) SPME fiber exposure times. TIC chromatograms are displayed in mirror-image format. *p*-Cresol emerges as the key odorant.

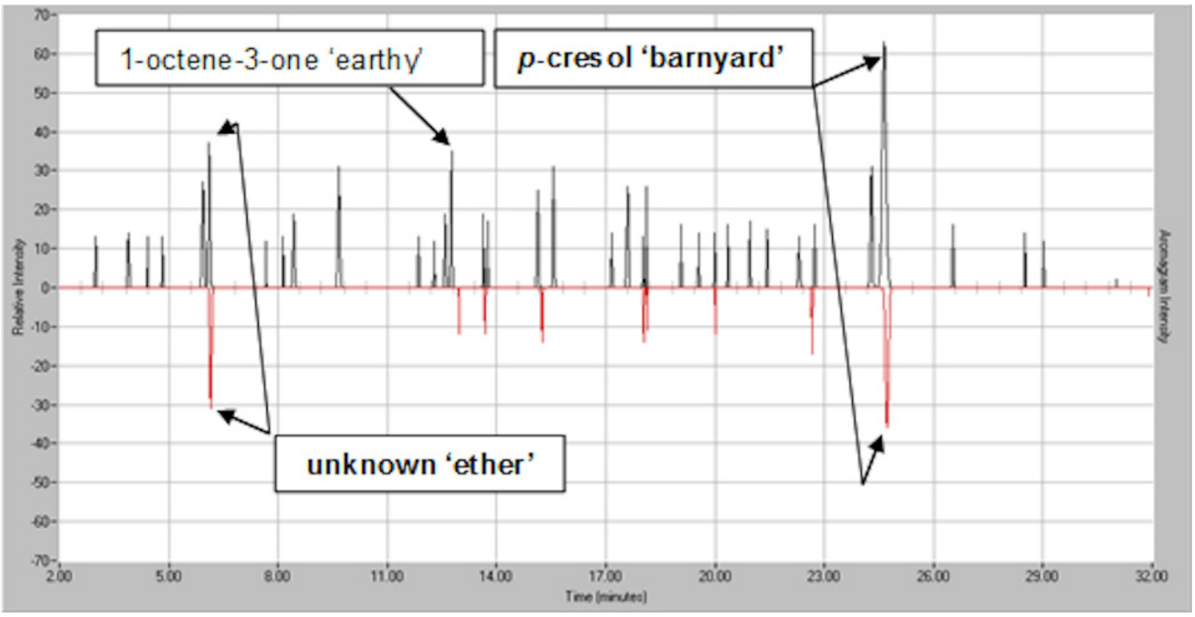

Figure S10. Serial dilution comparisons of the prairie verbena headspace odorants; aromagram odor profiles, generated by GC-Olfactometry. Contrasting odorant collections of 15 min (top) and 11 s (bottom) SPME fiber exposure times. Aromagrams are displayed in mirror-image format and illustrate how the *p*-cresol emerged as the key odorant. Impact priority odorants stand out from the multi-odorant background noise field.

The odorant prioritization suggests that the 'barnyard' odor at-distance-downwind is characteristic and is carried, almost exclusively, by the single, character-defining odorant, *p*-cresol. Key supporting evidence for this conclusion is:

- the 'barnyard' odor character, as perceived at the MDGC-MS-O sniff port for the isolated *p*-cresol peak, was virtually identical to the 'barnyard' smell perceived by the panelist at the odor frontal boundary (Figure S3);
- the *p*-cresol peak was the last detectable odorant response under the maximum dilution (i.e., 3 s SPME fiber exposure);
- the response to *p*-cresol as 'barnyard' was the only and last detectable odorant, despite the extremely brief, 3 s, exposure period; a condition known in SPME to bias against semi-VOCs.

Simplification was also reflected in an ~10-fold reduction in the VOC number and a corresponding 4-fold reduction in odorant 'notes' (Figures S8-S10), a process helpful in developing a simpler synthetic odorant subset. The odorant impact-priority subset was narrowed to:

- *p*-cresol (character-defining 'barnyard' or 'hog-truck' at the odor frontal boundary);
- –oxime isomers @6.0 min R.T. with their 'ether' or 'ketone' odor character;
- *trans*-calamanene @24.2 min with its 'spicy' 'baked bean' odor character;
- *alpha*-pinene with its 'pine oil' odor character;
- hyacinthin @18.0 min R.T. with its 'floral' odor character, and
- 1-octene-3-one with its 'earthy' or 'mushroom' odor character.

As shown in Table S1 below, the prairie verbena was only processed through the first 2 steps of the experimental process; as summarized in the Methods and Materials section of the main manuscript. However, this evidence would be sufficiently compelling to justify proceeding with subsequent Steps 3 to 5 (formal validation) and Step 6 (quantitative instrument-based monitoring protocol development) if this investigation had been tied to an actual environmental odor issue. These follow-up efforts would focus on *p*-cresol as the target 'character-defining' odorant for prioritizing odor monitoring and mitigation strategy purposes. In addition, expansion of the impact-priority subset was developed as follows:

Secondary priority impact was assigned to an odorant, eluting @6.0 min, which carries a distinct 'ether' or 'ketone' odor. Initial mass spectral fragmentation pattern data suggests that this unknown could be a series of –oxime isomers, but this prospect remains speculative at this juncture. Key supporting evidence for the secondary priority ranking includes: (1) a relatively strong response at the 11 s SPME fiber exposure condition; presenting with a lower odor intensity in comparison to the strong 'barnyard' response for *p*-cresol but a considerably higher odor intensity than the balance of the priority subset field and (2) the fact that this odorant continued to present with a relatively high-intensity response despite relatively lengthy SPME fiber exposure intervals; a condition potentially biasing against compounds carrying higher volatilities.

Third priority impact status was tentatively assigned to *trans*-calamanene for the 'spicy,' 'sweet' or 'baked bean' aroma note @24.2 min R.T. Key supporting evidence for this tertiary priority ranking includes: (1) *trans*-calamanene presents with relatively strong response at the 102 s SPME fiber exposure interval; a considerably lower odor intensity in comparison to the strong 'barnyard' response for *p*-cresol and 'ether' response for the unknown odorant @6.0 min R.T. but a considerably higher intensity in comparison to the remaining three components of the priority odorant subset and (2) the fact that the *trans*-calamanene 'spicy'

or ‘sweet’ odorant was among the last remaining priority odorant responses upon dilution, despite the relatively short 102 s exposure period; a condition known, under SPME sampling, to bias against compounds reflecting such limited volatility.

The ranking order of the priority subset's balance becomes much less definitive beyond the first three priority ranking positions and is therefore treated as a combined third-tier grouping. This being said, the incentive for the inclusion of the balance of the field includes: (1) *alpha*-pinene @ 9.6 min R.T. with its ‘pine oil’ odor character and hyacynthin, a ‘floral’ note @ 18.0 min R.T., both presenting with relatively strong responses at the 102 s fiber exposure interval and (2) 1-octene-3-one with its ‘earthy’ or ‘mushroom’ odor @ 12.8 min R.T. presenting with a relatively strong response between 102 s and 5 min fiber exposure intervals.

Table S1. Prairie verbena - Step 2 odor-match validation.

Panelist	Designation	Odor descriptor	Odor-match?	Match-source	Odor-match components
#1	lead investigator (D.W.W.)	‘barnyard’	yes	environment + chamber headspace + synthetic *p*-cresol odor-match formulation	environment + chamber headspace + GC-O + synthetic *p*-cresol odor-match formulation

MDGC-MS-O Contrasting downwind odorant prioritizations; the Central TX prairie verbena versus a North TX swine-barn:

The character-defining status of *p*-cresol relative to the frontal boundary odor-character of the prairie verbena clusters forms an interesting contrast to some of the more conventional, ‘agrarian’ sources of ‘barnyard’ odors (Table S2). Both of these profiles are very complex; as many as several hundred discrete VOCs, odorous and otherwise, have been previously identified as volatiles emissions from swine CAFOs [5]. Interestingly, from the standpoint of at-distance odor impact, it is evident in comparing the two listings that these two sources have little in common concerning VOC emission profiles despite sharing a virtually identical, at-distance odor character. Most importantly, they have no shared priority odorants beyond p-cresol, the single, highest impact and character-defining component for both. Likewise, the relative absence of expected odorants from a targeted odor source can be as telling as the identities of those odorants which are confirmed to be present. In that respect, it is believed noteworthy that the prairie verbena profiles are virtually free of significant odor contribution from reduced sulfurs, free-fatty acids, saturated amines, indolics, or phenolics (i.e., beyond *p*-cresol); all of which factor heavily in CAFO emission profiles. The absence of odor contribution from these odorous volatiles serves to magnify the impact significance of *p*-cresol and its singular correlation to the characteristic at-distance ‘barnyard’ odor, whether prairie verbena or swine barn sourced.

Table S2. Comparative impact-priority odorants; Prairie verbena vs. Swine Barn. Priority odorants **bolded, *common odorants*** are italicized and bolded.

Verbena Odorants *	Common priority odorants *	Swine Barn Odorants *
odor character = 'barnyard'	*'barnyard' odor*	*odor character = 'barnyard'*
***p*-cresol**	***p*-cresol**	***p*-cresol**
**-oxime unknown 'ether' @ 6.0 min**		**butyric acid**
**trans-calamanene (tentative ID)**		**isovaleric acid**
**alpha-pinene 'pine'**		**2-amino acetophenone**
**hycinthin 'floral' @ 18.0 min**		**4-ethyl phenol**
**1-octene-3-one 'earthy'**		**_quinazoline**
		**skatole**
		**indole**
Terpenes		*Sulfides*
beta-thujene		dimethyl trisulfide
camphene		methyl mercaptan
beta-myrcene		dimethyl sulfide
delta-3-carene		propyl mercaptan
I-phellandrene		dimethyl disulfide
alpha-terpinene		hydrogen sulfide
d-limonene		
o-cymene isomer		*Fatty Acids*
gamma-terpinene		valeric acid
delta-2-carene?		hexanoic acid
trans-ocimene		propanoic acid
alpha-copaene		acetic acid
alpha-farnesene		heptanoic acid
gamma-murrolene		
sabinene		*Amines*
beta-cymene		trimethylamine
		diethylamine
		1-pyrroline
Aromatics		*Aromatics*
guaiacol	***guaiacol***	***guaiacol***
benzaldehyde	***benzaldehyde***	***benzaldehyde***
benzyl isobutanoate		4-ethyl phenol
benzyl-2-methyl butyrate		phenol
benzyl-3-methyl butyrate		4-methyl-2-nitrophenol
benzyl alcohol		*p*-vinyl phenol
		benzoic acid
		phenyl acetic acid
		2-amino butophenone

Ketones		*Ketones*
acetone		2-octanone
2-butanone		6-methyl-5-heptene-2-one
		2-undecanone
		pentadecanone
		diacetyl
1-octene-3-one	***1-octene-3-one***	***1-octene-3-one***
Esters		
cis-carvyl acetate		*Aldehydes*
3-hexenyl-2-methyl butanoate		hexanal
methyl salicylate		nonanal
		methional
		undecanal
Alcohols		*Alcohols*
benzyl alcohol		1-octene-3-ol
		3-octanol
		1-heptene-3-ol
		trans-farnesol
		maltol
		geosmin
Miscellaneous		*Miscellaneous*
2-methyl furan	***2-methyl furan***	***2-methyl furan***
1,3-pentadiene		2-pentyl furan
2-methyl-1,3-pentadiene		dimethyl pyrazine
4,8-dimethyl-1,3,7-nonatriene		acetamide
1-methoxy-1,3,5-cycloheptatriene		4-methyl pyridine
tridecane		propanamide
		6-heptyltetrahydro-2H-pyran-2-one
		butanamide
		3-methyl-phenyl acetate
		phenyl ethyl alcohol
		pentamide
		2-pyrrolidinone
		hexadecane
		valerolactam
		5-methyl-2,4-imidazolidinedione

Notes: * many chemical identifications, beyond the impact-priority compounds, should be considered as tentative; they are the product of best-match efforts from Wiley and NIST mass spectral libraries matching. Many listed character-defining and character-impact odorants have been confirmed through on-instrument retention time and odor character matching.

Case Study #3: Virginia pepperweed

Initial odor assessment at the source and downwind

The first conscious introduction of the panelist (D.W.W.) to the smell was while mowing a lawn containing small clusters of Virginia pepperweed (Figure S11). At the source, the overall smell had the expected 'freshly cut grass', 'earthy' character. However, when the downwind odor frontal boundary was approached, a very familiar, intense, and distinct 'burnt match' odor character was detected. This 'burnt match' odor was recognizable due to past studies targeting various environmental odor sources [6].

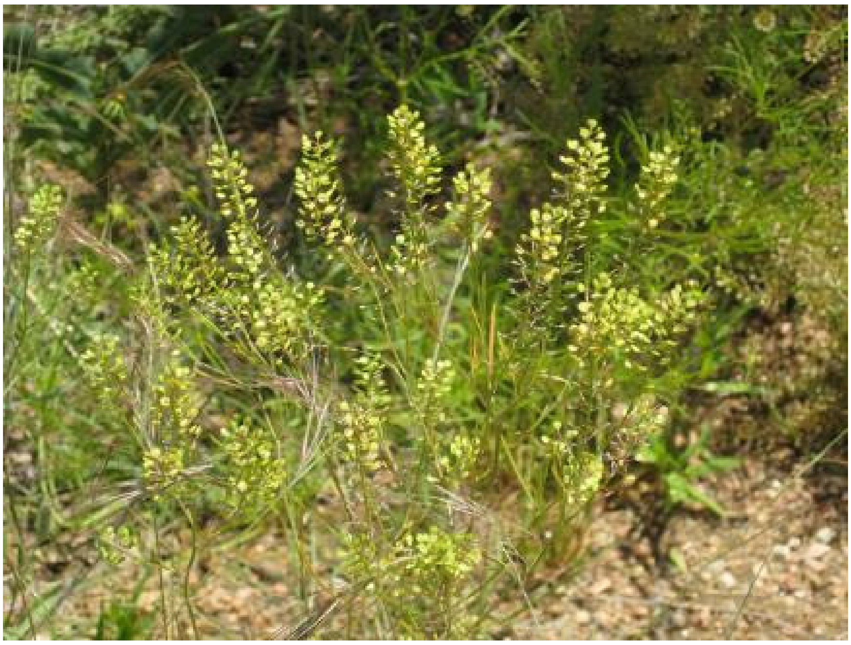

Figure S11. Virginia pepperweed; benzyl mercaptan 'burnt match' odor source (photo by D.W.W., [1]).

Working hypothesis based upon environmental odor assessment

The working hypothesis was: '*due to the remarkable similarity in downwind odor characteristics, there is assumed to be some chemical compound commonality between the priority odorant subsets for the Virginia pepperweed and some 'burnt match' odor sources*'. The driving question was:

- Is benzyl mercaptan correctly predicted as the character-defining odorant responsible for the 'burnt match' smell at the time of that first encounter and in advance of analytical confirmation?

MDGC-MS-O based environmental odorant prioritization

Follow-up MDGC-MS-O based odorant prioritization efforts confirmed the pre-analysis prediction that the character-defining odorant was traceable, dominantly to benzyl mercaptan (Figure S12). This prediction was significant; considering, that the panelist had to recognize and correctly identify a single odorous chemical from the hundreds, or perhaps thousands, of other possible odorous chemicals. The mechanical stressing of the plant was critical to release of the odorous VOCs, as shown below in the contrasting ms-SCAN TIC chromatograms profiling pristine versus crushed emission profiles.

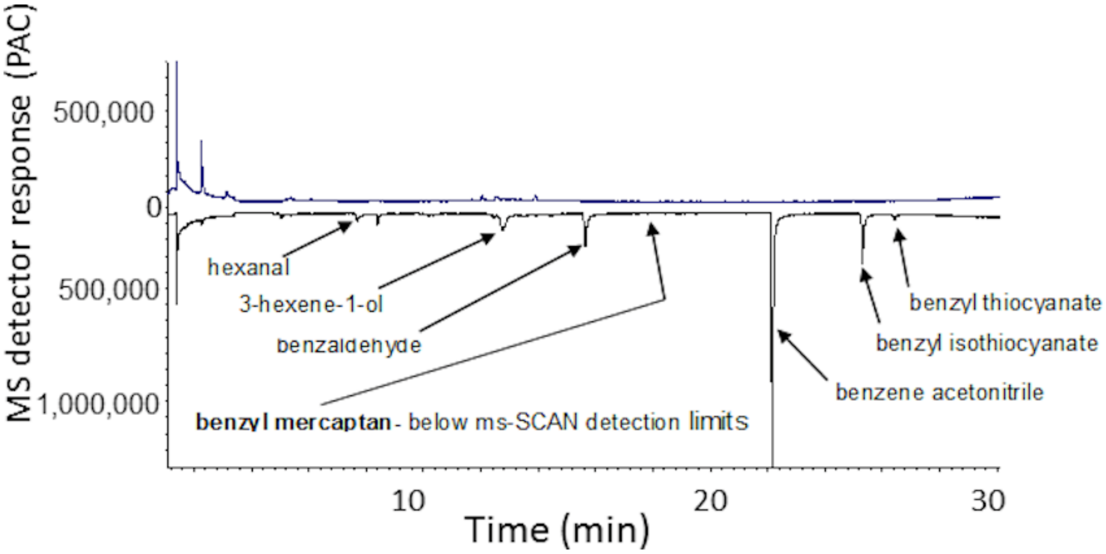

Figure S12. ms-SCAN TIC chromatogram of the Virginia pepperweed headspace volatiles. Total ion overview profiles are displayed in mirror-image format, reflecting pristine (top) versus crushed (bottom) states.

The overall odorous VOC emission profile from the crushed plant was complex, including prominent peaks of (1) benzyl thiocyanate; (2) benzyl isothiocyanate; (3) hexanal; (4) 3-hexene-1-ol; (5) benzaldehyde, and (6) benzene acetonitrile, among others (Figure S12). Interestingly, the character-defining benzyl mercaptan peak was not detectable under the initial ms-SCAN survey acquisition parameters. Therefore, the chemical identity confirmation for benzyl mercaptan was, initially, enabled by matching the known chromatographic retention time and known distinctive odor response for benzyl mercaptan at the olfactory detector. Subsequent MDGC-MS-O mass spectral library matching was able to confirm this initial assessment. The corresponding odor profiles for the pristine versus crushed are shown in Figure S13, below.

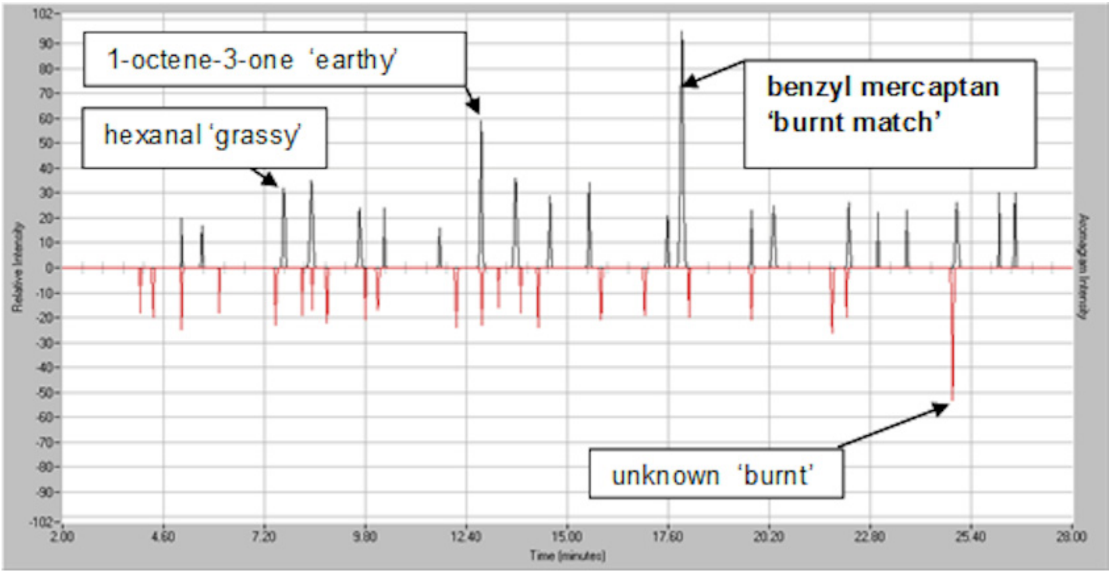

Figure S13. Comparison of odor profile aromagrams of the crushed (top) and pristine (bottom) Virginia pepperweed.

The apparent simplicity of benzyl mercaptan as 'impact-priority', responsible for the 'burnt match' odor at the frontal boundary, progressed to a distinctly different odor, of much greater complexity at the source. The overall odor near the opening of the 1 L glass vessel containing the crushed plants was perceived as 'grassy', 'herbaceous', and 'earthy'/'mushroom'. The overall impact-priority subset for the crushed Virginia pepperweed source was therefore proposed as (1) benzyl mercaptan (character-defining 'burnt-match' at the odor frontal boundary); (2) hexanal 'grassy'; (3) 1-octene-3-one 'earthy'; (4) 3-hexene-1-ol 'herbaceous'; (5) unknown 'ketone' @13.59 min R.T.; (6) benzaldehyde 'cherry'; (7) unknown 'burnt' @24.95 min R.T.; (8) methyl mercaptan 'fecal' and (9) hydrogen sulfide 'sewer'.

As shown in Table S3 below, the Virginia pepperweed was only processed through the first 3 steps of the experimental process; as summarized in the Methods and Materials section of the main manuscript. However, this evidence would be sufficiently compelling to justify proceeding through to Step 5 (formal validation) and Step 6 (quantitative instrument-based monitoring protocol development) if this investigation had been tied to an actual environmental odor issue. These follow-up efforts would focus on benzyl mercaptan as the target 'character-defining' odorant for prioritizing odor monitoring and mitigation assessment purposes.

Table S3. Virginia pepperweed - Step 3 odor-match validation.

Panelist	Designation	Odor descriptor	Odor-match?	Match-source	Odor-match components
#1	lead investigator (D.W.W.)	'burnt match'	yes	environment + chamber headspace + synthetic benzyl mercaptan odor-match formulation	environment + chamber headspace + GC-O + synthetic benzyl mercaptan odor-match formulation
#2	investigator #2 (A.I.)	'burnt'	yes	chamber headspace + synthetic benzyl mercaptan odor-match formulation	chamber headspace + GC-O + synthetic benzyl mercaptan odor-match formulation

References

1. Wright, D.W.; Koziel, J.A.; Parker, D.B.; Iwasinski, A.; Hartman, T.G.; Kolvig, P. Exploring Natural Models for the 'Rolling Unmasking Effect' of Downwind Odor Dispersion; Prairie Verbena, Prehensile-Tailed Porcupine and Virginia Pepperweed. *Preprints* **2018**, 2018030094, doi:10.20944/preprints201803.0094.v1.
2. Parker, D.; Rhoades, M.B.; Schuster, G.; Koziel, J.A.; Perschbacher-Buser, Z. Odor characterization at open-lot cattle feedyards using triangular forced-choice olfactometry. *Trans. ASABE* **2005**, *48*, 1527–1535.
3. Wright, D.W.; Nielsen, L.; Eaton, D.; Kuhrt, F.; Koziel, J.A.; Spinhirne, J.P.; Parker, D.B. Multidimensional GC-MS-olfactometry for identification and prioritization of malodors from confined animal feeding operations. *J. Agric. Food Chem.* **2005**, *53*, 8663–8672.
4. Wright, D.W.; Eaton, D.K.; Nielsen, L.T.; Kuhrt, F.W.; Koziel, J.A.; Parker, D.B. Improved, GC-olfactometry based, malodor assessment of swine CAFOs utilizing novel air sampling technologies. In Proceedings of the Air & Waste Management Association Symposium, Minneapolis, MN, USA, 21–24 June 2005.
5. Schiffman, S.S.; Bennett, J.L.; Raymer, J.H. Quantification of odors and odorants from swine operations in North Carolina. *Agricult. Forest. Meteorol* **2001**, *108*, 213–240.
6. Wright, D.; Wright, H.; Iwasinska, A.; Kuhrt, F.; Koziel, J.; Tippett-Mosby, L. Carthage bottoms area odor study; A Missouri test case for odorant prioritization as a prelude to instrument based downwind odor monitoring protocol development. In Proceedings of the 2008 ASABE Annual International Meeting, Providence, RI, USA, 29 June–2 July 2008.
